# *Selaginella* Genome Analysis – Entering the “Homoplasy Heaven” of the MADS World

**DOI:** 10.3389/fpls.2012.00214

**Published:** 2012-09-14

**Authors:** Lydia Gramzow, Elizabeth Barker, Christian Schulz, Barbara Ambrose, Neil Ashton, Günter Theißen, Amy Litt

**Affiliations:** ^1^Department of Genetics, Friedrich Schiller University JenaJena, Germany; ^2^Department of Biology, University of ReginaRegina, Canada; ^3^Department of Evolution and Biodiversity of Plants, Ruhr-University BochumBochum, Germany; ^4^The New York Botanical GardenBronx, NY, USA

**Keywords:** MADS-box genes, *Selaginella moellendorffii*, floral homeotic genes

## Abstract

In flowering plants, arguably the most significant transcription factors regulating development are MADS-domain proteins, encoded by Type I and Type II MADS-box genes. Type II genes are divided into the MIKC^C^ and MIKC* groups. In angiosperms, these types and groups play distinct roles in the development of female gametophytes, embryos, and seeds (Type I); vegetative and floral tissues in sporophytes (MIKC^C^); and male gametophytes (MIKC*), but their functions in other plants are largely unknown. The complete set of MADS-box genes has been described for several angiosperms and a moss, *Physcomitrella patens*. Our examination of the complete genome sequence of a lycophyte, *Selaginella moellendorffii*, revealed 19 putative MADS-box genes (13 Type I, 3 MIKC^C^, and 3 MIKC*). Our results suggest that the most recent common ancestor of vascular plants possessed at least two Type I and two Type II genes. None of the *S. moellendorffii* MIKC^C^ genes were identified as orthologs of any floral organ identity genes. This strongly corroborates the view that the clades of floral organ identity genes originated in a common ancestor of seed plants after the lineage that led to lycophytes had branched off, and that expansion of MIKC^C^ genes in the lineage leading to seed plants facilitated the evolution of their unique reproductive organs. The number of MIKC* genes and the ratio of MIKC* to MIKC^C^ genes is lower in *S. moellendorffii* and angiosperms than in *P. patens*, correlated with reduction of the gametophyte in vascular plants. Our data indicate that Type I genes duplicated and diversified independently within lycophytes and seed plants. Our observations on MADS-box gene evolution echo morphological evolution since the two lineages of vascular plants appear to have arrived independently at similar body plans. Our annotation of MADS-box genes in *S. moellendorffii* provides the basis for functional studies to reveal the roles of this crucial gene family in basal vascular plants.

## Introduction

Many important aspects of plant morphogenesis, including the specification of the architecture of both vegetative and generative parts, are controlled by members of multigene families that encode transcription factors. The most significant of these may be the MADS-box gene family. Members of this family are found in almost all eukaryotes but their numbers increased dramatically during plant evolution (Gramzow and Theißen, 2010). Since its expansion and diversification have been linked to the evolution of ontogenetic novelties in land plants (embryophytes; Theißen et al., 2000), studying evolution of the MADS-box gene family may illuminate the evolution of plant development.

Recently, the genome of the lycophyte *Selaginella moellendorffii* has been sequenced (Banks et al., 2011). *S. moellendorffii* represents the earliest evolutionary branch of vascular plants for which whole genome information is available; lycophytes bridge the gap between the green algal and bryophyte lineages, which branched off earlier in plant evolution, and the fern, gymnosperm, and angiosperm lineages (together referred to as euphyllophytes), which evolved after the lycophytes had branched off. Whole genome sequences of a number of green algal species and the bryophyte *Physcomitrella patens* (Rensing et al., 2008), as well as of several angiosperm species (e.g., The Arabidopsis Genome Initiative, 2000; Goff et al., 2002), are known. Whole genomes of ferns and gymnosperms have not yet been sequenced. Thus, the genome sequence of *S. moellendorffii* offers unique opportunities for understanding land plant evolution.

The life cycles of terrestrial plants probably evolved from the haplobiontic type characteristic of charophyte green algae (Graham et al., 2000; Lewis and McCourt, 2004) to one with a dominant gametophytic generation in bryophytes and to another with a dominant sporophytic generation in vascular plants (tracheophytes; Kenrick and Crane, 1997; Graham et al., 2000). As these changes occurred, the number of MADS-box genes appears to have increased from at least two genes in the most recent common ancestor (MRCA) of streptophytes (charophytes + land plants) via at least four genes in the MRCA of land plants to more than 10 genes in the MRCA of seed plants (spermatophytes; Gramzow and Theißen, 2010). In extant taxa, the differences are even more dramatic; whole genome sequences of chlorophyte green algae in some cases revealed only one MADS-box gene so far (Derelle et al., 2006; Merchant et al., 2007; Palenik et al., 2007), thus implying loss of at least one MADS-box gene; in contrast 26 MADS-box genes have been identified in the bryophyte *P. patens* (Rensing et al., 2008) and roughly about 100 MADS-box genes have been found in several angiosperm species (Martinez-Castilla and Alvarez-Buylla, 2003; Parenicova et al., 2003; Leseberg et al., 2006; Arora et al., 2007), indicating a massive increase in the number of gene family members during the evolution of land plants. Studying the complete complement of MADS-box genes in *S. moellendorffii* should provide more detailed insights into the expansion of the MADS-box gene family in land plants and its impact on the evolution of plant development and morphology.

MADS-domain transcription factors are characterized by the strongly conserved 58–60 amino acid DNA-binding MADS-domain. Two types of MADS-domain proteins, Types I and II, have been identified in essentially all eukaryotes including plants (Gramzow et al., 2010). Most if not all Type II proteins of streptophytes possess a second diagnostic region, the structurally conserved K domain (Alvarez-Buylla et al., 2000b; Martinez-Castilla and Alvarez-Buylla, 2003; Kaufmann et al., 2005) that is implicated in dimerization and higher order protein interactions (Ma et al., 1991; Melzer et al., 2009). The MADS and K domains are separated by a short intervening (I) domain, which also plays a role in protein interactions (Riechmann et al., 1996) even though its sequence is not strongly conserved. The C-terminal domain of MIKC-type MADS-domain proteins is highly variable, even among recently diverged sequences. There is evidence that it mediates higher order protein complex formation and, in specific cases, confers activities such as protein modification or transcription activation (Egea-Cortines et al., 1999; Yang et al., 2003; van Dijk et al., 2010). Because of their characteristic domain structure the Type II MADS-domain proteins of streptophytes are also termed MIKC-type proteins (Muenster et al., 1997).

Type I MADS-domain proteins do not have any conserved domains apart from the MADS-domain. In *Arabidopsis thaliana*, they have been subdivided into three groups, Mα, Mβ, and Mγ (Parenicova et al., 2003) but the phylogenetic status of these groups has not been rigorously tested. Previous studies suffer from limited sampling with respect to genes or species and low support for the topology of the trees that have been found (Parenicova et al., 2003; Nam et al., 2004; Leseberg et al., 2006; Arora et al., 2007). Also, comparatively little is known about the function of Type I genes in plants, even though they outnumber Type II genes in *A. thaliana* (De Bodt et al., 2003a; Martinez-Castilla and Alvarez-Buylla, 2003; Parenicova et al., 2003). A scattering of studies in *A. thaliana* has shown that some Type I genes are required for proper development of the female gametophyte (embryo sac) and/or endosperm and may play a role in post-zygotic lethality in interspecific hybrids (Kohler et al., 2003, 2005; Portereiko et al., 2006; Bemer et al., 2008; Colombo et al., 2008; Kang et al., 2008; Walia et al., 2009).

Type II MADS-domain proteins in plants are further subdivided into MIKC^C^ and MIKC* based on architectural and sequence differences (Henschel et al., 2002). About a dozen ancient clades of MIKC^C^-group MADS-box genes have been recognized while two classes of MIKC*-group genes, the P class and the S class, are distinguished in angiosperms (Nam et al., 2004) and ferns (Kwantes et al., 2011) based on their phylogenetic grouping. MIKC* genes are not well characterized but several appear to play important roles in the development of the male gametophyte (pollen) in angiosperms (Kofuji et al., 2003; Verelst et al., 2007a,b; Adamczyk and Fernandez, 2009; Zobell et al., 2010). In contrast, angiosperm MIKC^C^ genes have been studied in depth since many of them are key regulators of flowering time, floral organ identity, and fruit development (Schwarz-Sommer et al., 1990; Yanofsky et al., 1990; Huijser et al., 1992; Mandel et al., 1992; Pnueli et al., 1994; Michaels and Amasino, 1999; Ambrose et al., 2000; Lee et al., 2000; Honma and Goto, 2001; Becker et al., 2002; Becker and Theißen, 2003; Ferrario et al., 2003; Ditta et al., 2004; Pabón-Mora et al., 2012). Although gymnosperms possess orthologs representing most of the clades of MIKC^C^ floral developmental genes, phylogenetic analyses show that the genes identified from ferns, lycophytes, and mosses comprise other MIKC^C^ clades (Muenster et al., 1997; Hasebe et al., 1998; Mouradov et al., 1999; Sundstrom et al., 1999; Becker et al., 2000; Krogan and Ashton, 2000; Henschel et al., 2002; Svensson and Engstrom, 2002; Tanabe et al., 2003, 2005). Functional studies of MADS-box genes outside angiosperms are scarce: downregulation of three MIKC^C^ genes in *P. patens* resulted in diverse phenotypic effects in both the gametophyte and the sporophyte (Singer et al., 2007), and the liverwort gene, *MpMADS1* partially rescued *A. thaliana* MIKC* gene function in pollen development (Zobell et al., 2010).

Until now, our understanding of the evolution of this key family of developmental regulators has been derived from analyses of genomes of the phylogenetically distant moss and angiosperms. Here we identify 19 putative MADS-box genes in the genome of *S*. *moellendorffii*. We used sequence analysis and phylogeny reconstruction to classify the genes into the different classes of MADS-box genes. No orthologs belonging to any of the clades of floral organ identity genes could be identified; thus, the MIKC^C^ MADS-box genes in *S. moellendorffii* and seed plants seem to have diversified independently. Putative orthologs were found of Type I Mα genes, but not of Mβ or Mγ, and most of the *S. moellendorffii* Type I genes could not be classified into any of these three groups. Our findings provide a framework for more comprehensive investigations of the phylogeny of plant MADS-box genes and set the stage for studies on MADS-box gene functions in lycophytes.

## Materials and Methods

### Identification of genes and nomenclature

MADS-box genes in *S. moellendorffii* were annotated using the Genome Browser of the Joint Genome Institute (JGI, http://genome.jgi-psf.org/Selmo1/Selmo1.home.html). Sequences encoding MADS-domains were identified using the automated annotation provided by the JGI and by Hidden Markov Model (HMM) searches (Eddy, 1996) using the HMM profile described in Gramzow et al. (2010). The identified MADS-box genes were named using the prefix *SmMADS* and a number in order of discovery. Gene models provided by JGI’s automatic annotation pipeline were altered where comparisons with *S. moellendorffii* ESTs or with sequences and architectures of similar plant MADS-box genes suggested improvement. Splice sites conforming to plant consensus splice sites were used to define the boundaries of introns. In this study, Type I genes were initially assumed to comprise a single exon because most known Type I genes are single exon genes (De Bodt et al., 2003a; Parenicova et al., 2003). Sequences were extended, as needed, to incorporate a potential start codon. During the preparation of this paper, Kwantes et al. (2011) reported cDNA sequences of the three MIKC* genes in *S. moellendorffii* and we made slight changes to our models accordingly.

### Gene architecture analyses

To compare architectures of MIKC^C^ genes in *S. moellendorffii* with those in *A. thaliana*, one *A. thaliana* gene was selected from each of the 12 major clades and mean lengths of exons and introns were calculated using sequences from The Arabidopsis Information Resource (TAIR) database (Lamesch et al., 2012). Accession numbers are as follows: *PI* (At5g20240), *SEP1* (At5g15800), *AGL6* (At2g45650), *FUL* (At5g60910), *FLC* (At5g10140), *AG* (At4g18960), *AGL12* (At1g71692), *AGL15* (At5g13790), *AGL17* (At2g22630), *SOC1* (At2g45660), *SVP* (At2g22540), and *ABS* (At5g23260).

### Phylogeny reconstruction

Phylogenies of Type I and Type II MADS-box genes were reconstructed separately. The dataset of Type I MADS-box genes included all Type I genes of the first haplotype of *S. moellendorffii*, Type I sequences of *P. patens*, Type I sequences of gymnosperms as identified by BLAST searches (Altschul et al., 1990) and sequences of each of the three groups of Type I MADS-box genes, Mα, Mβ, and Mγ, that have been annotated in *A. thaliana* and *Oryza sativa* (Parenicova et al., 2003; Arora et al., 2007). Hence, all Type I genes of non-angiosperm plants available in GenBank are included in our phylogeny. As it is not clear whether MADS-box genes from green algae are monophyletic with Type I genes of plants, and as animal and fungal Type I genes are equally closely related to the Type I genes of plants (Alvarez-Buylla et al., 2000a; Gramzow et al., 2010), the Type I MADS-box gene *blistered*, encoding the *Drosophila melanogaster* Serum Response Factor (DSRF), was used as a representative of the outgroup.

All Type II MADS-box genes of the first haplotype of *S. moellendorffii*, Type II MADS-box genes of *P. patens*, and informative sequences of the major clades of MIKC^C^- and MIKC*-group MADS-box genes of *Marchantia polymorpha*, *Lycopodium annotinum*, *Gnetum gnemon*, *A. thaliana*, *Populus trichocarpa*, and *O. sativa*, were included in the dataset for Type II MADS-box genes. Additionally, Type II sequences of ferns were retrieved from GenBank using BLAST (Altschul et al., 1990). The Type II MADS-box gene *CgMADS1* of the streptophyte green alga *Chara globularis* was used as a representative of the outgroup.

Protein sequences inferred from Type I and Type II MADS-box genes were aligned separately using ProbCons (Do et al., 2005). Protein sequence alignments were reverse translated into nucleotide alignments using RevTrans (Wernersson and Pedersen, 2003). In the case of Type I genes, only the MADS-box regions, and in the case of Type II genes, the MADS- and the K-box regions, were used for phylogenetic analyses. The best substitution models for the nucleotide alignments were determined using the program Modeltest (Posada and Crandall, 1998). Bayesian phylogenies were reconstructed using the program MrBayes (Ronquist and Huelsenbeck, 2003). Analyses were done using the nucleotide alignments, where six million (Type I) and eight million (Type II) generations were run. We included, for the sake of completeness, posterior probabilities for all branches of phylogenies depicted in Figures 4, 5, and 6. However, for the purposes of interpretation and discussion we adopted a conservative approach based on branches supported by posterior probabilities ≥0.95, which are shown in bold in the figures.

## Results

### Identification of MADS-box genes in *Selaginella moellendorffii*

**Figure 1:**
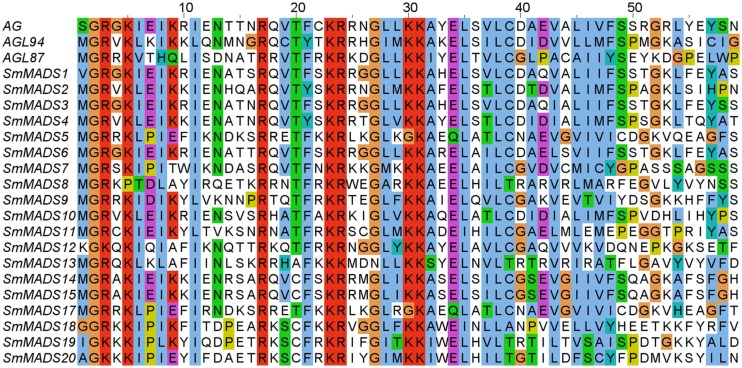
Alignment of MADS-domain sequences from three *A. thaliana* proteins (AG, AGL94, and AGL87, representing MIKC^C^, MIKC*, and Type I proteins, respectively) and the identified *S. moellendorffii* proteins (all other proteins). Amino acids are colored according to the ClustalW coloring scheme (Thompson et al., 1997) to highlight conservation. AG, SmMADS1, SmMADS12, SmMADS18, SmMADS19, and SmMADS20 do not start with Methionine as they have an N-terminal extension preceding the MADS-domain (not shown).

Examination of the genome of *S. moellendorffii* revealed 19 putative MADS-box genes. An alignment of representative MADS-domains of *A. thaliana* proteins and the MADS-domains of the identified *S. moellendorffii* proteins clearly corroborates the inclusion of the proteins encoded by these genes in the MADS-domain protein family (Figure 1). Two additional potential loci (*SmMADS16* and *SmMADS21*) are problematic and may actually represent misassembled versions of *SmMADS15* and *SmMADS20*, respectively. Furthermore, the two loci *SmMADS19* and *SmMADS20* overlap long terminal repeats and the MADS-box region contains repeat sequences. We have considered the latter two genes in our analyses but further investigation is needed to clarify their status as expressed genes. The MADS-boxes of *SmMADS1*, *SmMADS12*, *SmMADS18*, *SmMADS19*, and *SmMADS20* did not begin with a translation start codon so the sequences were extended in the 5′ direction to obtain the reading frame. The gene annotated as *SmMADS1* is quite similar (68% identical on the nucleotide level and 59% identical on the protein level) to *SrMADS1* which has been previously characterized in *S. remotifolia* (Tanabe et al., 2003).

**Figure 2:**
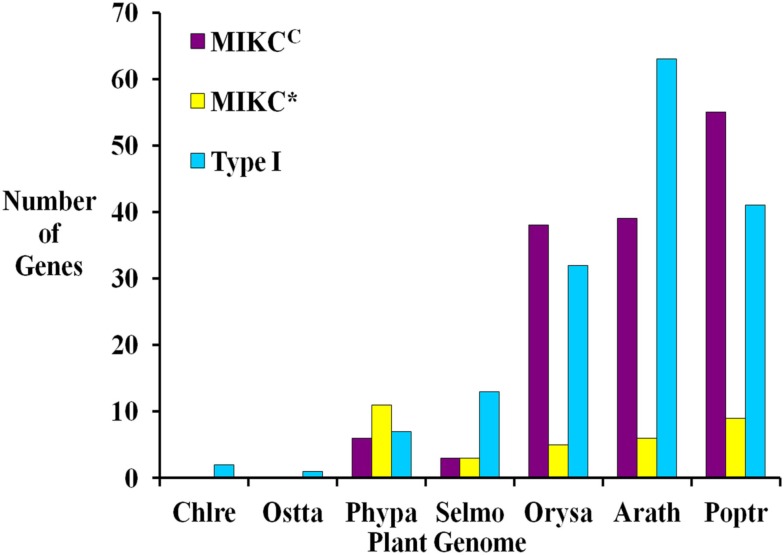
Comparison of the numbers of Type I, MIKC^C^, and MIKC* MADS-box genes in seven fully sequenced plant genomes including two green algae *Chlamydomonas reinhardtii* (Chlre; **Merchant et al., 2007), and *Ostreococcus tauri* (Ostta**; **Derelle et al., 2006), the moss *Physcomitrella patens* (Phypa**; **Rensing et al., 2008), *Selaginella moellendorffii* (Selmo), and the angiosperms *Oryza sativa* (Orysa**; **Arora et al., 2007), *Arabidopsis thaliana* (Arath**; **Parenicova et al., 2003) and *Populus trichocarpa* (Poptr**; **Leseberg et al., 2006)**. Note that elsewhere slightly different numbers of MADS-box genes have been reported for *A. thaliana* (De Bodt et al., 2003b; Kofuji et al., 2003) and *O. sativa* (Goff et al., 2002).

Based on sequence alignment and phylogenetic analysis, more than two-thirds (13) of the MADS-box genes appear to be Type I (Table 1), which represents a high fraction when compared to the gene content of other land plant genomes (Figure 2). All but one of these genes lack EST data; thus, their status as transcribed, functional genes remains to be verified. Of the six *S. moellendorffii* Type II sequences, three are MIKC^C^ and three are MIKC* (Table 1; Figure 2). Two of the MIKC^C^-group genes and one MIKC*-group gene are supported by EST data.

**Table 1:** The 19 *S. moellendorffii* MADS-box genes, designation as Type I, MIKC^C^, or MIKC*, number of coding exons, domains identified, existence of EST data, and GenBank Accession numbers for the two haplotypes.

Gene name	Type	Number of exons	Domains	EST evidence	Accession numbers
SmMADS1	MIKC^C^	8	NMIKC	Yes	XM_002977787
					XM_002979446
SmMADS2	MIKC*	12	MIKC	Yes	XM_002963649
					XM_002974738
SmMADS3	MIKC^C^	7	MIKC	Yes	XM_002984875
					XM_002985928
SmMADS4	MIKC*	11	MIKC	No	XM_002980998
					XM_002982473
SmMADS5	Type I	1	MC	Yes	XM_002961485
					XM_002971235
SmMADS6	MIKC^C^	7	MIKC	No	XM_002988269
					XM_002991371
SmMADS7	Type I	1	MC	No	XM_002970723
					XM_002991077
SmMADS8	Type I	1	MC	No	XM_002970254
					XM_002978417
SmMADS9	Type I	1	MC	No	XM_002970473
					XM_002978557
SmMADS10	MIKC*	10	MIKC	No	XM_002970396
					XM_002978468
SmMADS11	Type I	1	MC	No	XM_002966284
					XM_002978185
SmMADS12	Type I	2	NMC	No	XM_002969515
					XM_002970783
SmMADS13	Type I	1	MC	No	XM_002970468
					XM_002978551
SmMADS14	Type I (Mα)	1	MC	No	XM_002967770
					XM_002981654
SmMADS15	Type I (Mα)	1	MC	No	XM_002985619
SmMADS17	Type I	1	MC	No	XM_002979720
					XM_002985385
SmMADS18	Type I	2	NMC	No	XM_002973948
					XM_002983455
SmMADS19	Type I	2	NMC	No	XM_002973007
					XM_002984476
SmMADS20	Type I	1	NMC	No	XM_002973124
					XM_002976491

*Domains are abbreviated as follows: N, N-terminal domain; M, MADS-domain; I, Intervening domain; K, Keratin-like domain; C, C-terminal domain*.

### Gene architecture

**Figure 3:**
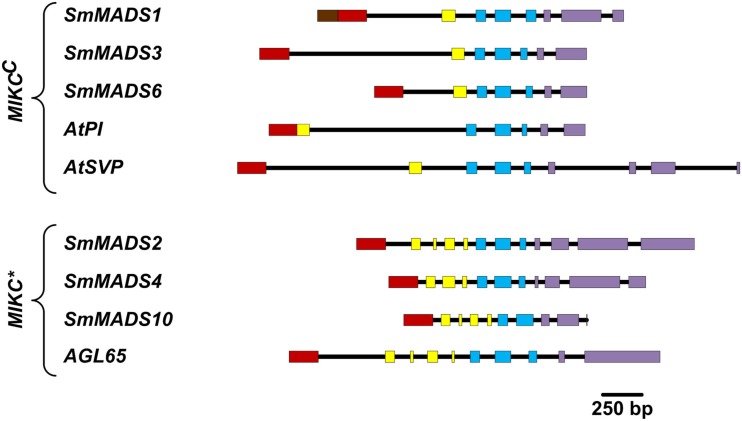
**Comparison of gene architectures of *S. moellendorffii* Type II genes with selected Type II genes from *A. thaliana*, which were chosen to illustrate the range of exon-intron structures**. Only protein-coding exons were considered. Exons are represented by rectangles where the coloring scheme follows Henschel et al. (2002; N-terminal region – brown; MADS-box – red; I-region – yellow; K-box – blue; C-terminal region – purple) and introns are represented by black lines.

We have analyzed the number of protein-coding exons in *S*. *moellendorffii* and *A. thaliana* MADS-box genes. As EST data is lacking for most *S*. *moellendorffii* MADS-box genes it is impossible to analyze the complete number of exons since some exons solely comprised of UTR have probably been missed during gene annotation. Three Type I genes are comprised of two exons (Table 1). All other Type I genes are single exon genes. MIKC^C^ genes in *S*. *moellendorffii* contain the same mean number of exons (7.3) as the exemplar MIKC^C^ genes from *A. thaliana* (7.3) although the range is narrower in *S*. *moellendorffii* (7–8) than in *A. thaliana* (6–9; Figure 3). The mean total exonic length of *MIKC^C^* genes in *S*. *moellendorffii* (789 bp) is slightly greater than that of the exemplar *A. thaliana* MIKC^C^ genes (712 bp). In contrast, mean combined intronic length for *S*. *moellendorffii* MIKC^C^ genes (998 bp) is less than half that for *A. thaliana* MIKC^C^ genes (2090 bp).

MIKC* genes in *S. moellendorffii* contain a greater average number of exons (11) than in *A. thaliana* (10). Additionally, the range is larger (10–12) for the *S. moellendorffii* genes than for the *A. thaliana* genes, which comprise 10 exons each. The mean combined exonic length (1044 bp) in *S. moellendorffii* MIKC* genes is approximately the same as in *A. thaliana* MIKC* genes (1022 bp). The mean combined intronic length in *S. moellendorffii* MIKC* genes (601 bp) is considerably less than that of *A. thaliana* MIKC* genes (1070 bp).

### Duplication of MADS-box genes

The *S*. *moellendorffii* genome does not appear to have been subjected to whole genome duplication (Banks et al., 2011). *SmMADS21*, a gene encoding a phosphoethanolamine-binding protein (PEBP), and a kiwellin-like gene are linked within about 5 kb and are separated by approximately 20 kb from a group of genes that includes *SmMADS20*, a PEBP pseudogene, and a second kiwellin-like gene, also contained within approximately 5 kb of DNA. Each gene/pseudogene of a pair is highly similar in nucleotide sequence to the other. Our gene model for *SmMADS21* contains an N-terminal extension. However, an alignment of the nucleotide sequences of *SmMADS20* and *SmMADS21* suggests that a single nucleotide has been inserted near the 5′ end of the ancestral sequence, shifting the reading frame in *SmMADS21* and requiring that the sequence be extended at the 5′ end to incorporate a start codon. *SmMADS20* and *SmMADS21* are 97% identical at the amino acid level. The kiwellin-like sequences are 95% identical at the amino acid level. Thus, if *SmMADS21* is considered a *bona fide* gene, *SmMADS20* and *SmMADS21* probably arose from duplication of a section of DNA at least 5 kb long. There is no other case in the *S. moellendorffii* genome in which a MADS-box gene and two or more adjacent genes in one location are highly similar to three or more adjacent genes in another location.

**Figure 4:**
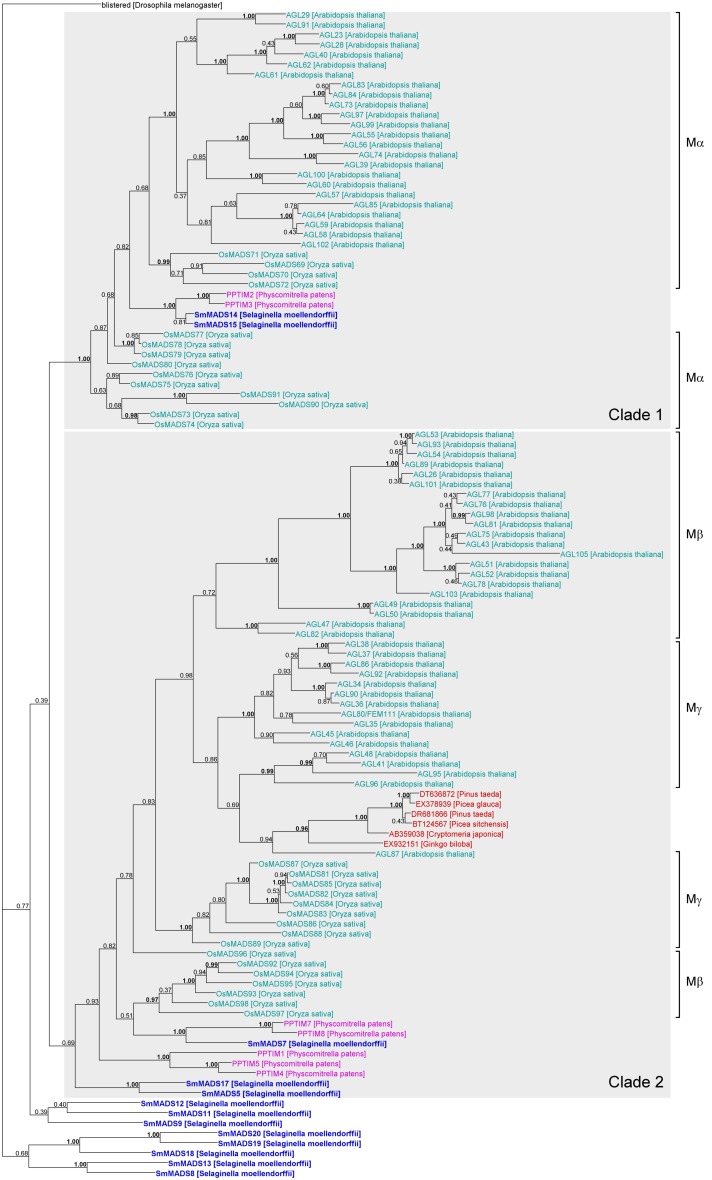
**Phylogenetic tree of Type I MADS-box genes constructed using MrBayes**. The groups of Type I genes as annotated in *A. thaliana* (Parenicova et al., 2003) and *O. sativa* (Arora et al., 2007) are indicated by brackets on the right. Two possible ancient clades of land plant Type I genes are indicated by gray boxes. Gene names are color coded as follows: light blue – angiosperm genes, red – gymnosperm genes, dark blue – lycophyte genes, purple – bryophyte genes. Genes from *S. moellendorffii* are highlighted in bold writing. Posterior probabilities are indicated on the branches. Those ≥0.95 are shown in bold.

Three Type I genes (*SmMADS8*, *SmMADS9*, *SmMADS13*) are located on scaffold 14 and the outermost of this trio of genes are separated by about 98 kb. *SmMADS8* and *SmMADS13* cluster together in the phylogenetic tree (see below and Figure 4). However, they are less than 40% identical at the amino acid level, suggesting that if they are the result of an ancient tandem duplication, they have diverged considerably in sequence and been physically separated by insertions of DNA. *SmMADS9*, which is situated between the other two MADS-box genes and is less than 25% identical to them, may have been among the genes inserted. *SmMADS7* and *SmMADS12* are located on the same scaffold, but since they are separated by more than 1 Mb of DNA and they are not in the same phylogenetic cluster, the linkage may be coincidental.

### Phylogenies

We have constructed separate alignments and phylogenies for Type I and Type II MADS-box genes from *S. moellendorffii* together with informative sets of MADS-box genes from other completely sequenced plant genomes as well as MADS-box genes from species at critical phylogenetic positions in the green plant lineage. Although the monophyly of Type I genes has not been strongly supported, we chose to analyze all non-MIKC genes together to investigate membership of *S. moellendorffii* genes in defined gene clades.

Our phylogeny of Type I genes reveals that while a subdivision into clades of Mα, Mβ, and Mγ genes works reasonably well for Type I genes from *A. thaliana*, many genes from *O. sativa* appear to have been misassigned (Arora et al., 2007; Figure 4). In fact, our phylogeny suggests that there are two ancient clades of Type I genes, one containing all Mα genes and another one containing all Mβ and Mγ genes (labeled “Clade 1” and “Clade 2,” respectively in Figure 4). The genes *SmMADS14* and *SmMADS15* of *S. moellendorffii* as well as *PPTIM2* and *PPTIM3* of *P. patens* belong to a clade which only additionally contains Mα genes of *A. thaliana* and *O. sativa* (Clade 1, Figure 4). Thus, an Mα gene probably already existed in the MRCA of mosses and vascular plants about 500 MYA (million years ago) (Zimmer et al., 2007). Three *S. moellendorffii* Type I genes, *SmMADS5*, *SmMADS7*, and *SmMADS17* and five MADS-box genes from *P. patens*, *PPTIM1*, *PPTIM4*, *PPTIM5*, *PPTIM7*, and *PPTIM8*, can be assigned to a clade which is otherwise comprised of all Mβ and Mγ genes of *A. thaliana* and *O. sativa* and some MADS-box genes from gymnosperms (Clade 2, Figure 4). Hence, the MRCA of mosses and vascular plants probably also possessed an ancestral gene to the Mβ and Mγ genes of *A. thaliana*. The other *S. moellendorffii* Type I genes appear to have diversified independently. They form two clades at the base of the phylogeny comprised of three and five genes, respectively. In our phylogenies, there is no evidence of additional Type I genes in the MRCA of vascular plants, suggesting the MRCA of vascular plants also possessed at least two Type I genes.

**Figure 5:**
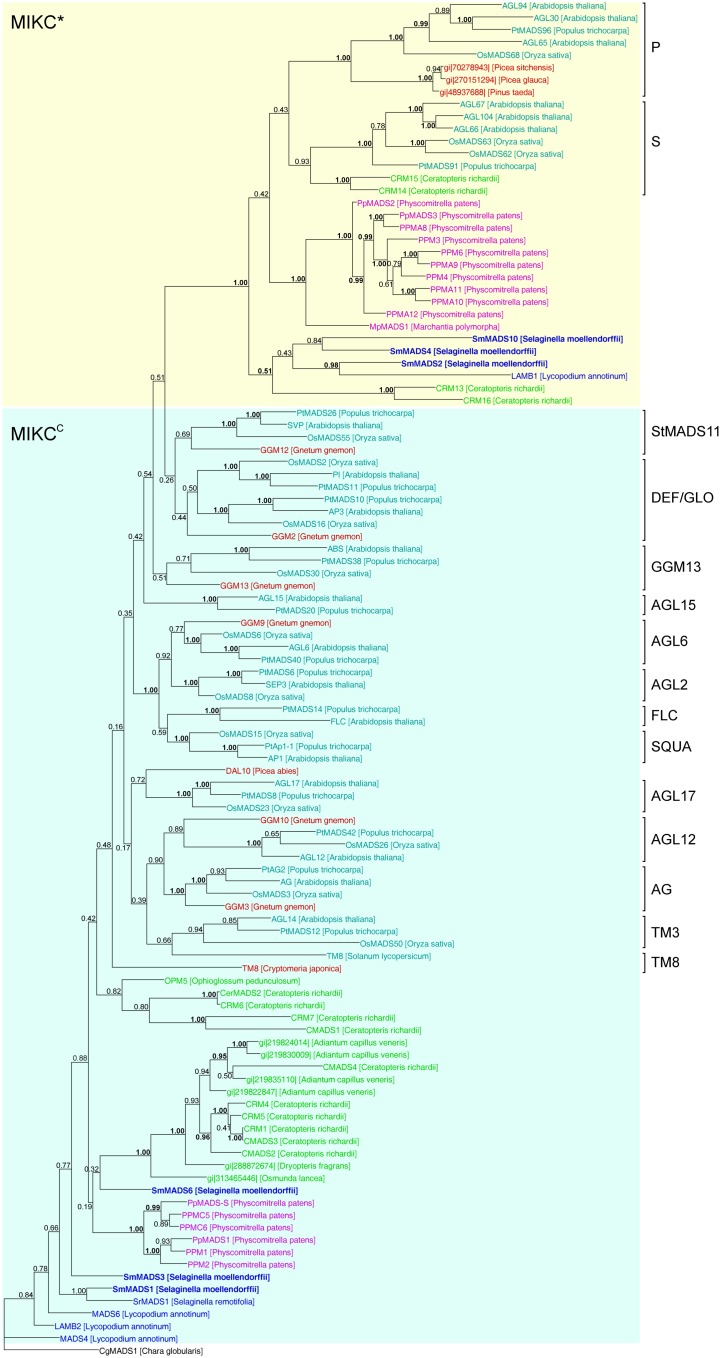
**Phylogenetic tree of Type II MADS-box genes constructed using MrBayes**. The different clades of Type II MADS-box genes are indicated by brackets on the right. MIKC*- and MIKC^C^-group genes are indicated by yellow and light blue shading, respectively. Gene names are color coded as in Figure 4 and green – fern genes. Genes from *S. moellendorffii* are highlighted in bold writing. Posterior probabilities are indicated on the branches. Those ≥0.95 are shown in bold.

**Figure 6:**
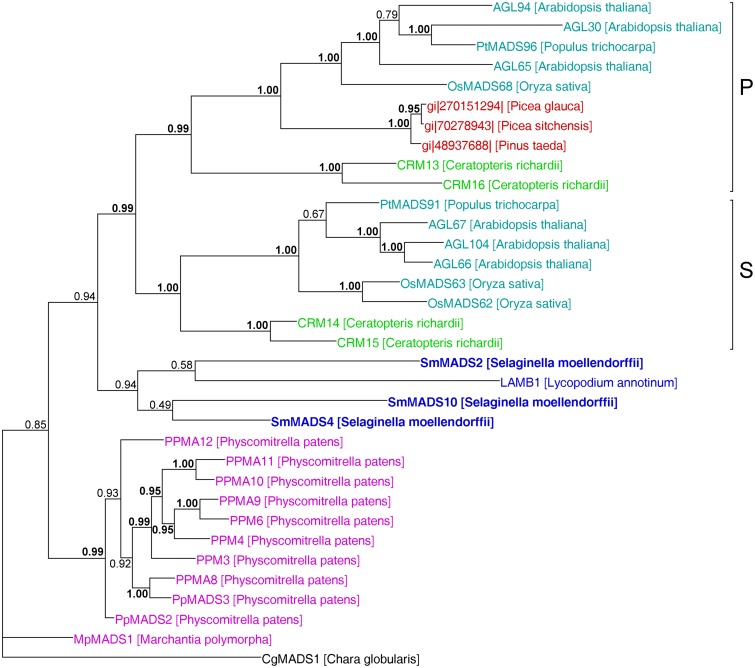
MrBayes phylogeny of MIKC* genes. The clades P and S are indicated by brackets on the right. Gene names are color coded as in Figures 4 and 5. Genes from *S. moellendorffii* are highlighted in bold writing. Posterior probabilities are indicated on the branches. Those ≥0.95 are shown in bold.

In our analysis of Type II MADS-box genes, MIKC* genes consistently form a clade that includes *P. patens*, *S. moellendorffii*, fern, gymnosperm, and angiosperm genes (Figure 5). Thus, at least one MIKC* gene probably existed in the MRCA of mosses and vascular plants. Our phylogeny suggests that the *S. moellendorffii* MIKC* gene *SmMADS2* is orthologous to *LAMB1* of *Lycopodium annotinum*. These two genes cluster together with the other two *S. moellendorffii* MIKC* genes, *SmMADS4*, and *SmMADS10*, and *CRM13* and *CRM16* of the fern *Ceratopteris richardii*. This clade is basal to all other MIKC* genes in our phylogeny, whereas *CRM13* and *CRM16* were identified as P class genes by Kwantes et al. (2011). The MIKC* genes of *P. patens* cluster together, suggesting an independent diversification of these genes in mosses. The MIKC* gene of the liverwort *M. polymorpha* is sister to the *P. patens* clade; this bryophyte clade is sister to the clade of P class genes of angiosperms and gymnosperms as well as to the clade of S class genes of angiosperms and ferns. As some posterior probabilities were quite low for the clade of MIKC* genes, we constructed a separate phylogeny for these genes (Figure 6). This separate phylogeny has much higher posterior probabilities. Here, the P class genes, including *CRM13* and *CRM16*, and the S class genes form sister clades. Basal to these clades is a clade comprised of all the known MIKC* genes of lycophytes. All MIKC* genes from *P. patens* form a clade which is basal to all MIKC* genes from vascular plants, and *MpMADS1* of *M*. *polymorpha* is the basal-most gene in our phylogeny.

MIKC^C^-group MADS-box genes are not monophyletic in our phylogeny because the MIKC*-group genes are nested within them (Figure 5). The only gene which has been previously characterized from a *Selaginella* species, *SrMADS1* (Tanabe et al., 2003), clusters together with the gene we annotated as *SmMADS1* in *S. moellendorffii*. However, in our phylogeny these two genes do not form a monophyletic clade with *LAMB2*, *MADS4*, and *MADS6* of *L. annotinum* as found by Tanabe et al. (2003); rather they form a separate moderately supported early diverging branch, as does a second MIKC^C^ gene of *S. moellendorffii*, *SmMADS3*. The earlier branching of some lycophyte genes relative to the *P. patens* genes is in contrast to the land plant phylogeny and may be due to problems in resolving the deep branching of divergent taxa. Consistent with green plant phylogeny, another clade of fern MIKC^C^ genes occupies an intermediate position between the genes of lycophytes and *P. patens* and the major clades of MIKC^C^ genes of seed plants. Importantly, orthologous relationships between floral organ identity genes from angiosperms and any of the MIKC^C^ genes from *S. moellendorffii* were not detected. Our phylogeny suggests that the MRCA of mosses and vascular plants also possessed an ancestral MIKC^C^ gene. As with Type I genes, there is no evidence for an expansion of Type II genes from the MRCA of mosses and vascular plants to the MRCA of vascular plants; thus the genome of the latter probably encoded at least two Type II genes, i.e., one MIKC* and one MIKC^C^ gene.

## Discussion

### Number of MADS-box genes compared to other species

Only one MADS-box gene has been found in some chlorophyte green algal species and in each of three charophycean green algae, close relatives of land plants (Tanabe et al., 2005; Derelle et al., 2006; Merchant et al., 2007; Palenik et al., 2007; Figure 2). However, the MRCA of green plants probably possessed at least two MADS-box genes as it has been shown that the two types (I and II) of MADS-box genes had already been established in the MRCA of extant eukaryotes, and both types of genes are present in land plants (Alvarez-Buylla et al., 2000a; Gramzow et al., 2010). These findings strongly suggest that at least some lineages of green algae have lost one type of MADS-box gene. In contrast, flowering plants possess approximately 100 MADS-box genes (e.g., 107 in *A. thaliana*, 75 in *O. sativa*; Parenicova et al., 2003; Arora et al., 2007; Figure 2). Consequently, there is a high probability that a considerable expansion of the MADS-box gene family happened during the about 480 million years (MY) of land plant evolution (Zimmer et al., 2007). The extent and nature of this expansion varies between different plant lineages. In the genomes of *S. moellendorffii*, *A. thaliana*, *P. trichocarpa*, and *O. sativa*, Type I genes make up at least 40% of MADS-box genes while only about 20% of MADS-box genes in *P. patens* are Type I (Parenicova et al., 2003; Leseberg et al., 2006; Arora et al., 2007; Rensing et al., 2008). Hence, there appears to have been a greater relative expansion of Type I genes in the tracheophyte lineage (lycophytes + ferns + seed plants) than in the moss lineage. Only a few *A. thaliana* Type I MADS-box genes have been functionally characterized (Kohler et al., 2003; Portereiko et al., 2006; Yoo et al., 2006; Bemer et al., 2008; Colombo et al., 2008; Kang et al., 2008; Steffen et al., 2008). Since these have roles in the development of the female gametophyte as well as the embryo and the seed, their proliferation in tracheophytes may be related to the evolution of reproductive structures. However, it has also been shown that Type I genes have faster rates of gene gain and loss than Type II genes (Nam et al., 2004). Therefore, the observed ratios of Type I to Type II genes may be due to these random processes rather than having a functional basis. The overall significance of plant Type I MADS-box genes has not been resolved. While some have been assigned definite functions, it has been proposed that many of them possess features characteristic of selfish genetic elements (De Bodt et al., 2003a).

Within the Type II group, *P. patens* has twice as many MIKC* as MIKC^C^ genes, *S. moellendorffii* has equal numbers, and *A. thaliana*, *P. trichocarpa*, and *O. sativa* possess approximately 6–7 times as many MIKC^C^ as MIKC* genes (Figure 2; Parenicova et al., 2003; Leseberg et al., 2006; Arora et al., 2007; Rensing et al., 2008). MIKC* MADS-box genes are expressed in the gametophytes of *A. thaliana* and mosses (Verelst et al., 2007a,b; Zobell et al., 2010) and are assumed to play a role in gametophytic development. *P. patens* and other bryophytes have a gametophyte-dominant life-cycle whereas the gametophytic stage is highly reduced in tracheophytes. Hence, the expansion of MIKC* genes in bryophytes and their much lower proportion, relative to MIKC^C^ genes, in tracheophytes may be correlated, at least in part, with the different life cycles of these plants.

### Characteristics of MADS-box genes in *S. moellendorffii*

The mean total exonic length of Type II genes in *S. moellendorffii* is similar to that of *A. thaliana* Type II genes. In contrast, the mean combined intronic length of *S. moellendorffii* Type II genes is slightly more than half that of *A. thaliana* Type II genes. In general, introns in *S. moellendorffii* are around 35% shorter than those in *A. thaliana* (Banks et al., 2011). Thus, the difference in intron lengths between Type II genes in *S*. *moellendorffii* and *A. thaliana* is greater than the genome-wide trend. The significance of retention of less than average proportions of intronic sequence in MADS-box genes in *S. moellendorffii* compared with *A. thaliana* is unknown but it is possible that lack of conservation of important regulatory elements was involved.

MIKC^C^ genes in *S*. *moellendorffii* have a more narrow range of numbers of exons than *A. thaliana* MIKC^C^ genes. It is possible that similarity in architecture has resulted from duplication of MIKC^C^ genes within the *S*. *moellendorffii* lineage. However, this similarity may also be an artifact of the gene annotation process in which *S*. *moellendorffii* MADS-box gene models were constructed by comparison with each other as well as with MADS-box genes from other plants. Altogether, the number of protein-coding exons is similar in *S. moellendorffii* and *A. thaliana* Type II genes. Hence, as has been recognized for *P. patens* and *A. thaliana* genes (Henschel et al., 2002), the exon-intron structures of *S. moellendorffii* Type II genes exhibit considerable conservation despite about 400 MY of independent evolution.

Our study has revealed scant information regarding the nature of the duplication events that have produced the complement of *S. moellendorffii* MADS-box genes, which contrasts with the situation in angiosperm species (Parenicova et al., 2003; Arora et al., 2007). Only for *SmMADS20* and *SmMADS21* are there indications of an ancestral MADS-box gene being duplicated as part of the duplication of a substantial section of DNA (at least 5 kb). Explanations of this duplication event based on transposon activity have not been excluded. However, given the relatively close locations of the duplicated DNA segments, we suggest tentatively that unequal crossing over, perhaps facilitated by nested repeats noted in this region, is the most plausible explanation. However, *SmMADS21* may also represent a misassembled version rather than a duplicate of *SmMADS20*. The scarcity of evidence that the MADS-box gene family in *S. moellendorffii* has expanded through recent duplication events suggests that some mechanisms involved in expansion of the MADS family have not been as active in *S. moellendorffii* as in some other plants.

### Age of MADS-box gene clades

Our phylogenies of Type I and Type II MADS-box genes reveal few supported clades comprising genes from both seed plants (spermatophytes) and non-seed plants (Figures 4 and 5). For Type I genes, there seem to be two ancient clades of land plant genes (“Clade 1” and “Clade 2” in Figure 4). One clade is comprised of the Mα genes of *A. thaliana* and *O. sativa* and two genes each of *S. moellendorffii* and *P. patens*. The other clade includes the Mβ and Mγ genes of *A. thaliana* and *O. sativa*, three genes of *S. moellendorffii*, and five genes of *P. patens*. The conservation of these genes in several major lineages of land plants may indicate an ancient and potentially conserved function, possibly in female gametophytic or embryonic development as indicated by recent data from *A. thaliana* (Kohler et al., 2003; Yoo et al., 2006; Bemer et al., 2008; Colombo et al., 2008; Kang et al., 2008; Steffen et al., 2008). However, Type I MADS-box genes also exhibit features of selfish genetic elements (De Bodt et al., 2003a) and their conservation may only be an indication of their ability to preserve themselves. Under this hypothesis the Type I genes that have a function in development may have been recruited to play that role by their host plants secondarily.

Our phylogenetic analyses also indicate that the Mβ and Mγ genes as annotated previously may not be monophyletic. Studies of MADS-box genes in *O. sativa* and *P. trichocarpa* had indicated paraphyly of Mβ genes already (Leseberg et al., 2006; Arora et al., 2007). Hence, the subdivision of Type I genes into groups requires further investigation.

Our separate MIKC* phylogeny (Figure 6) suggests that the duplication which gave rise to the classes S and P happened in a common ancestor of euphyllophytes after lycophytes had branched off. Our phylogeny differs in some aspects from the one obtained by Kwantes et al. (2011). While MIKC* genes from bryophytes are sister to S class genes in the phylogeny obtained by Kwantes et al. (2011), the bryophyte genes are basal in our phylogeny. The genes from lycophytes are basal-most in the phylogeny of Kwantes et al. (2011), while they form a clade in our phylogeny that branches off after the genes from bryophytes have branched off. The differences in the phylogenies may be explained by the use of different methods for phylogeny reconstruction and different taxon sampling. Kwantes et al. (2011) used a neighbor joining method to reconstruct the phylogeny, whereas we used a Bayesian method, which generally results in more reliable phylogenies (Holder and Lewis, 2003). The support values in our phylogeny are higher and our phylogeny fits better to the species phylogeny of land plants, indicating a more reliable reconstruction, even though Kwantes et al. (2011) included more sequences in their analysis.

MIKC^C^ genes of *S. moellendorffii* take up a quite basal position in our phylogeny of Type II genes. Importantly, orthologous relationships between floral organ identity genes from angiosperms and any of the MIKC^C^ genes from *S. moellendorffii* were not detected. Previous analyses found orthologs of some floral organ identity genes in gymnosperms, but had suggested that these genes are absent from non-seed plants such as mosses and ferns (e.g., Muenster et al., 1997; Hasebe et al., 1998; Mouradov et al., 1999; Sundstrom et al., 1999; Krogan and Ashton, 2000; Theißen et al., 2000, 2001; Becker et al., 2002; Henschel et al., 2002; Svensson and Engstrom, 2002; Becker and Theißen, 2003). However, because whole-genome data were lacking, the possibility that those orthologs in non-seed plants simply escaped detection had not been ruled out. The analysis of the genome of *P. patens* (Rensing et al., 2008) already confirmed the absence of floral organ identity gene orthologs from a moss species. Our analyses of the *S. moellendorffii* genome now suggest that the clades of floral homeotic genes and other MIKC^C^-type genes known from angiosperms originated not earlier than the separation of the lycophyte lineage from the lineage that led to ferns (*sensu lato*) and seed plants 400–450 MYA (Zimmer et al., 2007). Whether there are such genes in ferns remains to be seen but appears to us unlikely, given that several independent attempts to isolate them have failed (e.g., Muenster et al., 1997; Hasebe et al., 1998; Münster et al., 2002).

### Implications for the evolution of reproductive structures

With the evidence at hand indicating that bryophytes and lycophytes do not possess clear orthologs to the floral organ identity genes of seed plants, and under the assumption that ferns also do not have orthologs, two possibilities remain regarding evolution of the developmental programs controlling formation of reproductive structures in land plants. In the first, it is conceived that the development of reproductive structures in both seed and non-seed plants is controlled by orthologous MADS-box genes. However, for this to be true, the sequences of the corresponding MADS-box genes must have diversified so extensively that orthologous relationships cannot be recovered by standard phylogenetic analyses. The second, and probably more plausible, possibility is that different (i.e., non-orthologous) genetic factors are involved in the developmental programs of reproductive structures in seed and non-seed plants. Assuming that MADS-box genes control developmental processes also in lycophytes, this would imply that in lycophytes, and at least in some euphyllophytes (specifically, gymnosperms and angiosperms) major developmental programs evolved independently. This independent origin of MADS-box gene-based developmental programs would be correlated, and even causally linked, to the independent origin of some remarkably similar morphological structures, such as seed-like structures and seeds, in the two sister groups lycophytes and euphyllophytes, respectively (Friedman, 2011). The prospects of studying the homoplasious origin of structures in lycophytes independent of that in euphyllophytes (ferns and allies, gymnosperms, angiosperms) have been termed “homoplasy heaven” (Friedman, 2011). In keeping with this, one may argue that a comparative study of MADS-box genes in the sister groups of euphyllophytes and in lycophytes allows one to enter the homoplasy heaven of the MADS world.

Which genes regulate the development of the sporophyte, especially reproductive development, in non-seed plants, including lycophytes, remains to be elucidated. In seed plants, this development is controlled mainly by specific clades of MIKC^C^-type genes (Theißen et al., 2000; Becker and Theißen, 2003). According to what has been said above, it seems likely that these genes were recruited for their new functions soon after their divergence into different clades in a common ancestor of extant seed plants after the lineage that led to extant ferns had branched off 300–400 MYA (Zimmer et al., 2007).

### Concluding summary and outlook

In our study we identified 19 MADS-box genes in *S. moellendorffii* of which 13 are Type I and 6 are Type II. We find that the MRCA of land plants likely had at least two Type I and two Type II genes and confirm the general trend of MADS-box gene family expansion in land plants. Given the ubiquitous presence of MADS-box genes in embryophytes together with what we know already about their important roles in the development and evolution of spermatophytes, it is clear that we should now afford a high priority to functional analyses of the complete MADS-box gene complement discerned within the *S. moellendorffii* genome. It will be interesting to discover, for example, whether any of the *S. moellendorffii* genes encode a reproductive function as has been shown for some MIKC^C^ genes in *P. patens* (Quodt et al., 2007; Singer et al., 2007) and appears likely for charophycean algae (Tanabe et al., 2005).

## Conflict of Interest Statement

The authors declare that the research was conducted in the absence of any commercial or financial relationships that could be construed as a potential conflict of interest.
